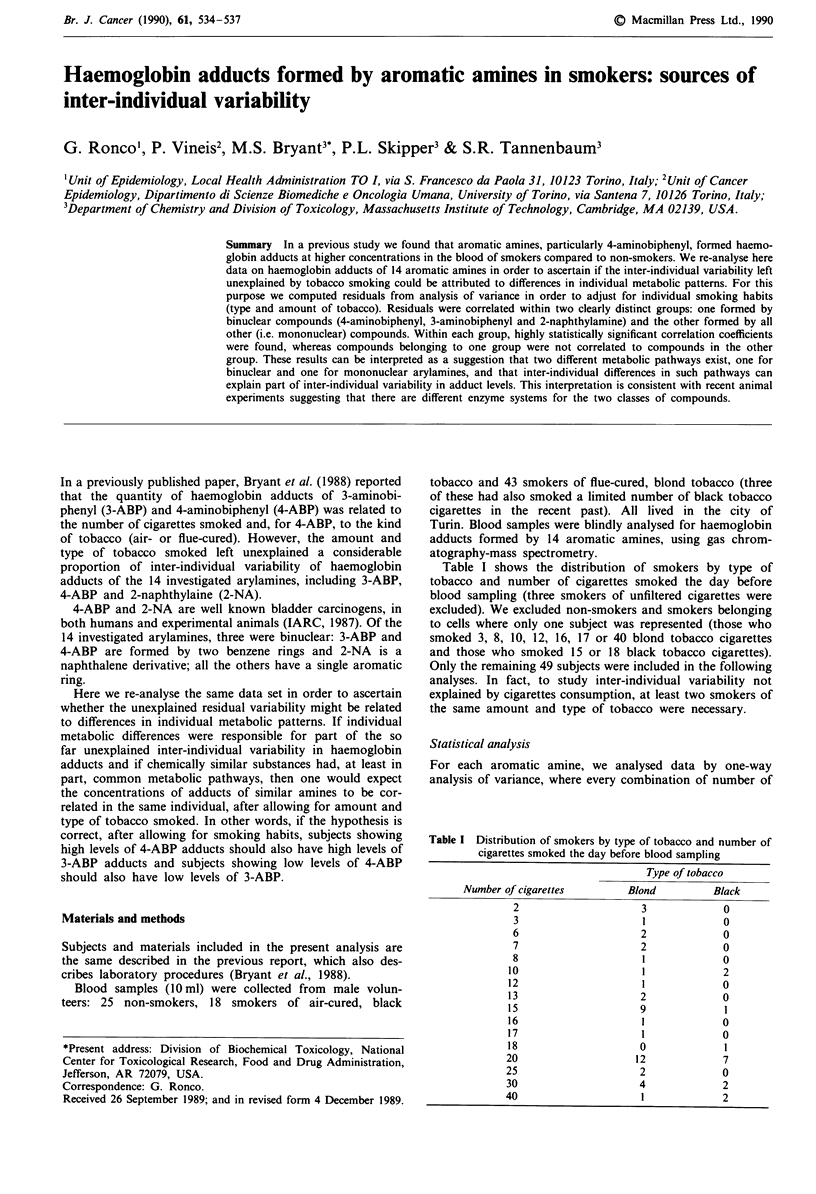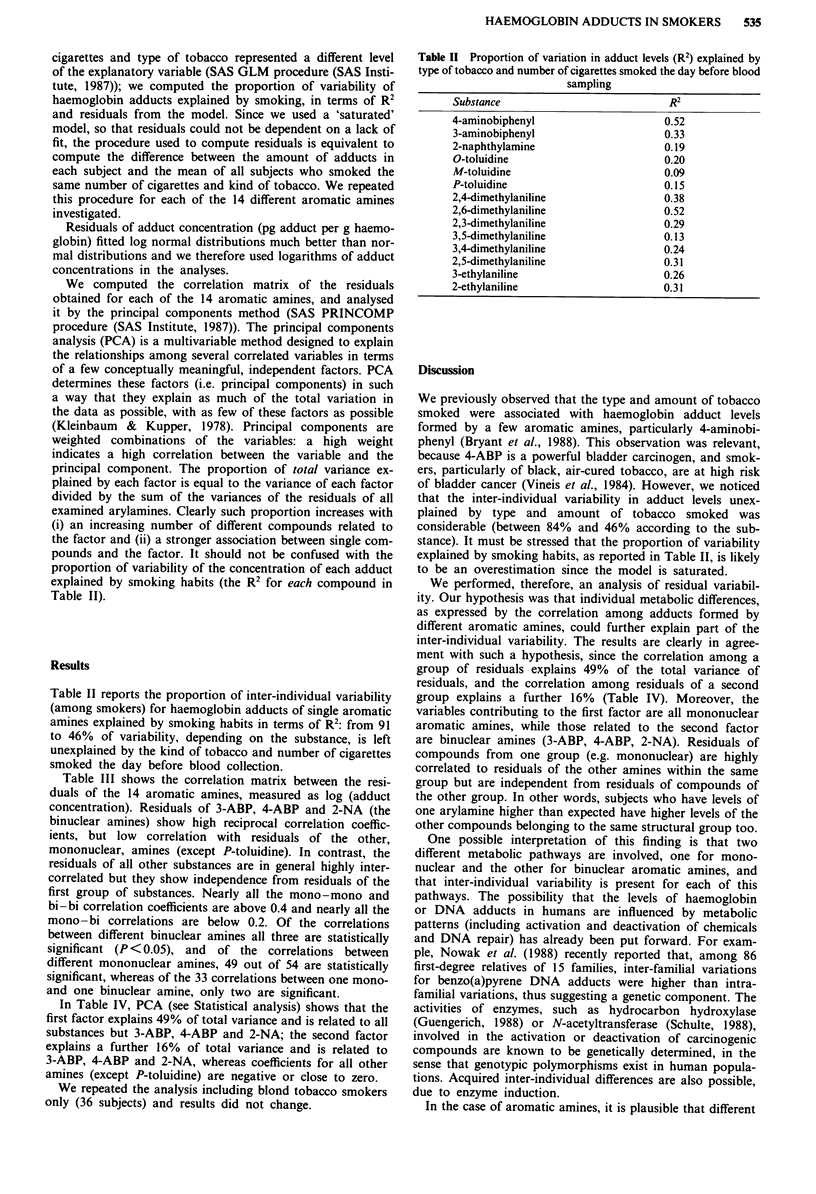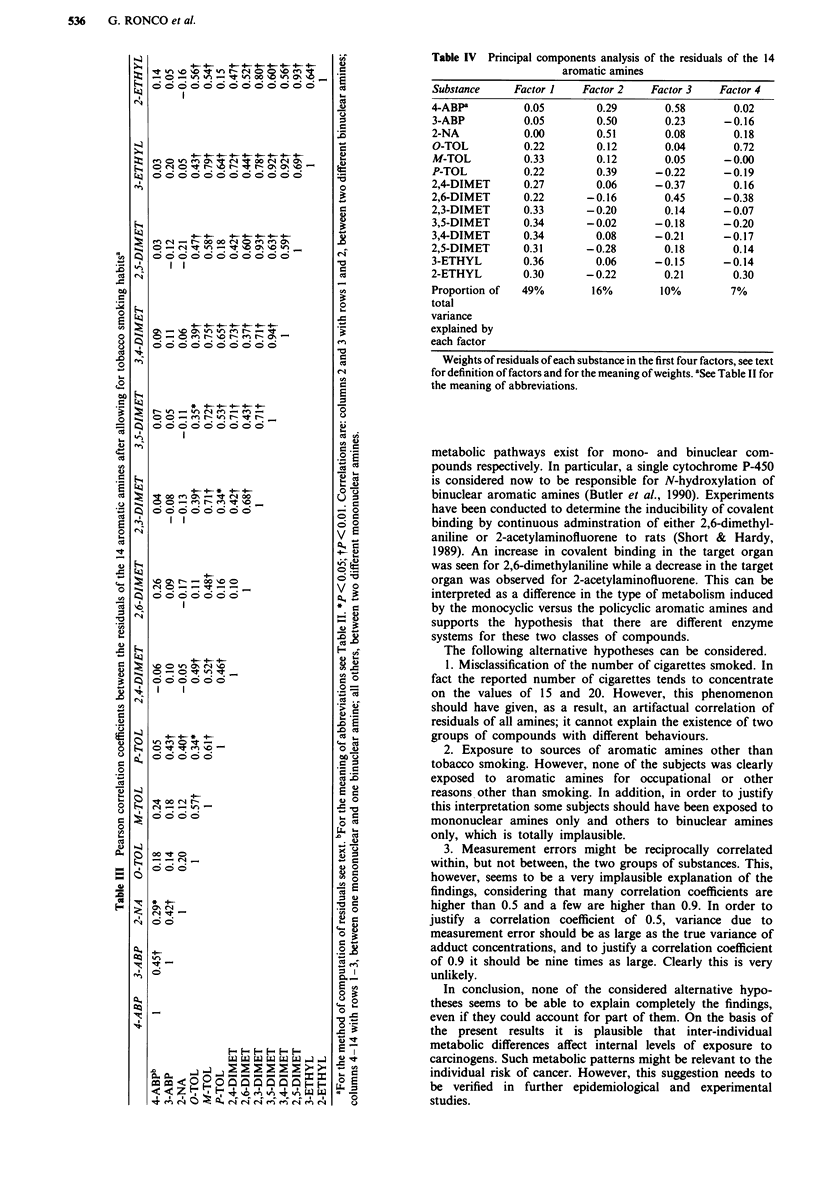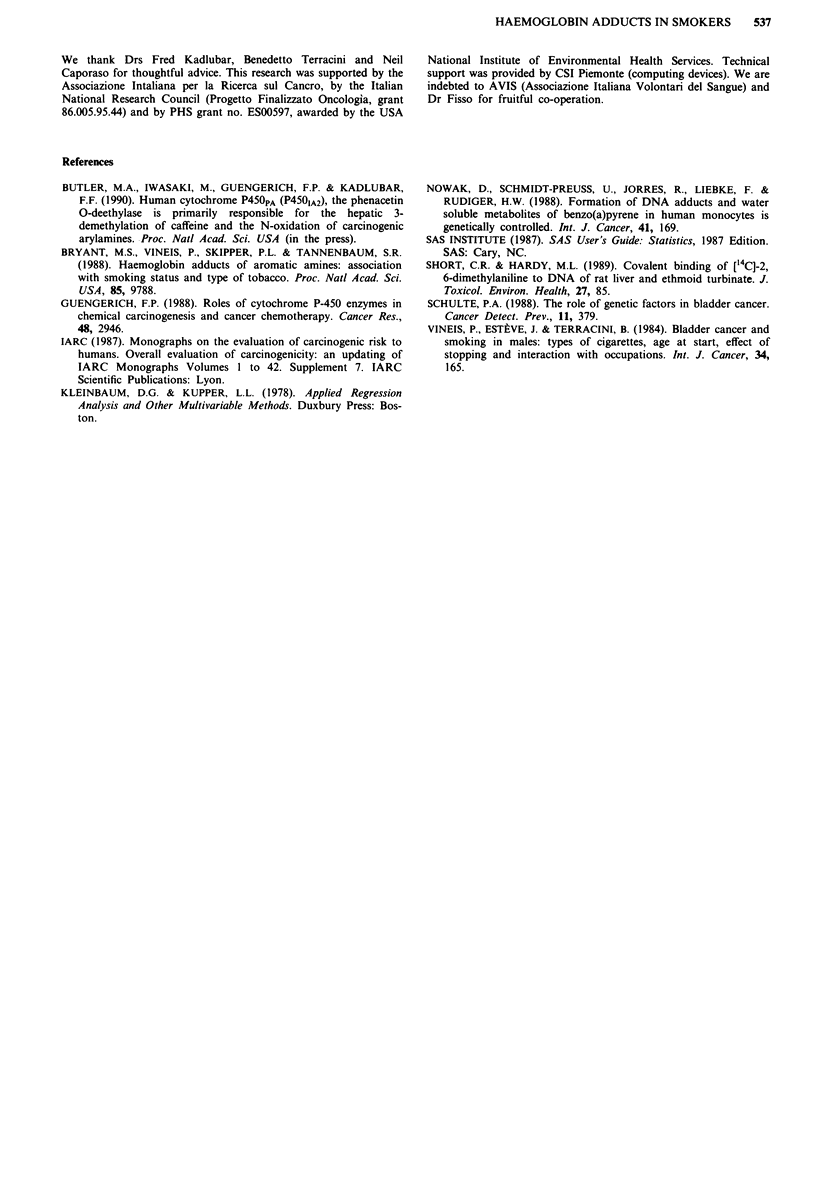# Haemoglobin adducts formed by aromatic amines in smokers: sources of inter-individual variability.

**DOI:** 10.1038/bjc.1990.120

**Published:** 1990-04

**Authors:** G. Ronco, P. Vineis, M. S. Bryant, P. L. Skipper, S. R. Tannenbaum

**Affiliations:** Unit of Epidemiology, Local Health Administration TO I, Torino, Italy.

## Abstract

In a previous study we found that aromatic amines, particularly 4-aminobiphenyl, formed haemoglobin adducts at higher concentrations in the blood of smokers compared to non-smokers. We re-analyse here data on haemoglobin adducts of 14 aromatic amines in order to ascertain if the inter-individual variability left unexplained by tobacco smoking could be attributed to differences in individual metabolic patterns. For this purpose we computed residuals from analysis of variance in order to adjust for individual smoking habits (type and amount of tobacco). Residuals were correlated within two clearly distinct groups: one formed by binuclear compounds (4-aminobiphenyl, 3-aminobiphenyl and 2-naphthylamine) and the other formed by all other (i.e. mononuclear) compounds. Within each group, highly statistically significant correlation coefficients were found, whereas compounds belonging to one group were not correlated to compounds in the other group. These results can be interpreted as a suggestion that two different metabolic pathways exist, one for binuclear and one for mononuclear arylamines, and that inter-individual differences in such pathways can explain part of inter-individual variability in adduct levels. This interpretation is consistent with recent animal experiments suggesting that there are different enzyme systems for the two classes of compounds.


*Br. J. Cancer* (1990), **61**, 534–537 © Macmillan Press Ltd., 1990

### Haemoglobin adducts formed by aromatic amines in smokers: sources of inter-individual variability

G. Ronco[1], P. Vineis[2], M.S. Bryant[3*], P.L. Skipper[3] & S.R. Tannenbaum[3]

[1]*Unit of Epidemiology, Local Health Administration TO I, via S. Francesco da Paola 31, 10123 Torino, Italy;* [2]*Unit of Cancer Epidemiology, Dipartimento di Scienze Biomediche e Oncologia Umana, University of Torino, via Santena 7, 10126 Torino, Italy;* [3]*Department of Chemistry and Division of Toxicology, Massachusetts Institute of Technology, Cambridge, MA 02139, USA.*

**Summary** In a previous study we found that aromatic amines, particularly 4-aminobiphenyl, formed haemoglobin adducts at higher concentrations in the blood of smokers compared to non-smokers. We re-analyse here data on haemoglobin adducts of 14 aromatic amines in order to ascertain if the inter-individual variability left unexplained by tobacco smoking could be attributed to differences in individual metabolic patterns. For this purpose we computed residuals from analysis of variance in order to adjust for individual smoking habits (type and amount of tobacco). Residuals were correlated within two clearly distinct groups: one formed by binuclear compounds (4-aminobiphenyl, 3-aminobiphenyl and 2-naphthylamine) and the other formed by all other (i.e. mononuclear) compounds. Within each group, highly statistically significant correlation coefficients were found, whereas compounds belonging to one group were not correlated to compounds in the other group. These results can be interpreted as a suggestion that two different metabolic pathways exist, one for binuclear and one for mononuclear arylamines, and that inter-individual differences in such pathways can explain part of inter-individual variability in adduct levels. This interpretation is consistent with recent animal experiments suggesting that there are different enzyme systems for the two classes of compounds.

In a previously published paper, Bryant *et al.* (1988) reported that the quantity of haemoglobin adducts of 3-aminobiphenyl (3-ABP) and 4-aminobiphenyl (4-ABP) was related to the number of cigarettes smoked and, for 4-ABP, to the kind of tobacco (air- or flue-cured). However, the amount and type of tobacco smoked left unexplained a considerable proportion of inter-individual variability of haemoglobin adducts of the 14 investigated arylamines, including 3-ABP, 4-ABP and 2-naphthylaine (2-NA).

4-ABP and 2-NA are well known bladder carcinogens, in both humans and experimental animals (IARC, 1987). Of the 14 investigated arylamines, three were binuclear: 3-ABP and 4-ABP are formed by two benzene rings and 2-NA is a naphthalene derivative; all the others have a single aromatic ring.

Here we re-analyse the same data set in order to ascertain whether the unexplained residual variability might be related to differences in individual metabolic patterns. If individual metabolic differences were responsible for part of the so far unexplained inter-individual variability in haemoglobin adducts and if chemically similar substances had, at least in part, common metabolic pathways, then one would expect the concentrations of adducts of similar amines to be correlated in the same individual, after allowing for amount and type of tobacco smoked. In other words, if the hypothesis is correct, after allowing for smoking habits, subjects showing high levels of 4-ABP adducts should also have high levels of 3-ABP adducts and subjects showing low levels of 4-ABP should also have low levels of 3-ABP.

### Materials and methods

Subjects and materials included in the present analysis are the same described in the previous report, which also describes laboratory procedures (Bryant *et al.*, 1988).

Blood samples (10 ml) were collected from male volunteers: 25 non-smokers, 18 smokers of air-cured, black tobacco and 43 smokers of flue-cured, blond tobacco (three of these had also smoked a limited number of black tobacco cigarettes in the recent past). All lived in the city of Turin. Blood samples were blindly analysed for haemoglobin adducts formed by 14 aromatic amines, using gas chromatography-mass spectrometry.

Table I shows the distribution of smokers by type of tobacco and number of cigarettes smoked the day before blood sampling (three smokers of unfiltered cigarettes were excluded). We excluded non-smokers and smokers belonging to cells where only one subject was represented (those who smoked 3, 8, 10, 12, 16, 17 or 40 blond tobacco cigarettes and those who smoked 15 or 18 black tobacco cigarettes). Only the remaining 49 subjects were included in the following analyses. In fact, to study inter-individual variability not explained by cigarettes consumption, at least two smokers of the same amount and type of tobacco were necessary.

### *Statistical analysis*

For each aromatic amine, we analysed data by one-way analysis of variance, where every combination of number of

**Table I** Distribution of smokers by type of tobacco and number of cigarettes smoked the day before blood sampling

| | *Type of tobacco* | |
|---|---|---|
| *Number of cigarettes* | *Blond* | *Black* |
| 2 | 3 | 0 |
| 3 | 1 | 0 |
| 6 | 2 | 0 |
| 7 | 2 | 0 |
| 8 | 1 | 0 |
| 10 | 1 | 2 |
| 12 | 1 | 0 |
| 13 | 2 | 0 |
| 15 | 9 | 1 |
| 16 | 1 | 0 |
| 17 | 1 | 0 |
| 18 | 0 | 1 |
| 20 | 12 | 7 |
| 25 | 2 | 0 |
| 30 | 4 | 2 |
| 40 | 1 | 2 |

*Present address: Division of Biochemical Toxicology, National Center for Toxicological Research, Food and Drug Administration, Jefferson, AR 72079, USA.
Correspondence: G. Ronco.
Received 26 September 1989; and in revised form 4 December 1989.

cigarettes and type of tobacco represented a different level of the explanatory variable (SAS GLM procedure (SAS Institute, 1987)); we computed the proportion of variability of haemoglobin adducts explained by smoking, in terms of $R^2$ and residuals from the model. Since we used a 'saturated' model, so that residuals could not be dependent on a lack of fit, the procedure used to compute residuals is equivalent to compute the difference between the amount of adducts in each subject and the mean of all subjects who smoked the same number of cigarettes and kind of tobacco. We repeated this procedure for each of the 14 different aromatic amines investigated.

Residuals of adduct concentration (pg adduct per g haemoglobin) fitted log normal distributions much better than normal distributions and we therefore used logarithms of adduct concentrations in the analyses.

We computed the correlation matrix of the residuals obtained for each of the 14 aromatic amines, and analysed it by the principal components method (SAS PRINCOMP procedure (SAS Institute, 1987)). The principal components analysis (PCA) is a multivariable method designed to explain the relationships among several correlated variables in terms of a few conceptually meaningful, independent factors. PCA determines these factors (i.e. principal components) in such a way that they explain as much of the total variation in the data as possible, with as few of these factors as possible (Kleinbaum & Kupper, 1978). Principal components are weighted combinations of the variables: a high weight indicates a high correlation between the variable and the principal component. The proportion of *total* variance explained by each factor is equal to the variance of each factor divided by the sum of the variances of the residuals of all examined arylamines. Clearly such proportion increases with (i) an increasing number of different compounds related to the factor and (ii) a stronger association between single compounds and the factor. It should not be confused with the proportion of variability of the concentration of each adduct explained by smoking habits (the $R^2$ for *each* compound in Table II).

### Results

Table II reports the proportion of inter-individual variability (among smokers) for haemoglobin adducts of single aromatic amines explained by smoking habits in terms of $R^2$: from 91 to 46% of variability, depending on the substance, is left unexplained by the kind of tobacco and number of cigarettes smoked the day before blood collection.

Table III shows the correlation matrix between the residuals of the 14 aromatic amines, measured as log (adduct concentration). Residuals of 3-ABP, 4-ABP and 2-NA (the binuclear amines) show high reciprocal correlation coefficients, but low correlation with residuals of the other, mononuclear, amines (except *P*-toluidine). In contrast, the residuals of all other substances are in general highly intercorrelated but they show independence from residuals of the first group of substances. Nearly all the mono–mono and bi–bi correlation coefficients are above 0.4 and nearly all the mono–bi correlations are below 0.2. Of the correlations between different binuclear amines all three are statistically significant ($P<0.05$), and of the correlations between different mononuclear amines, 49 out of 54 are statistically significant, whereas of the 33 correlations between one mono- and one binuclear amine, only two are significant.

In Table IV, PCA (see Statistical analysis) shows that the first factor explains 49% of total variance and is related to all substances but 3-ABP, 4-ABP and 2-NA; the second factor explains a further 16% of total variance and is related to 3-ABP, 4-ABP and 2-NA, whereas coefficients for all other amines (except *P*-toluidine) are negative or close to zero.

We repeated the analysis including blond tobacco smokers only (36 subjects) and results did not change.

**Table II** Proportion of variation in adduct levels ($R^2$) explained by type of tobacco and number of cigarettes smoked the day before blood sampling

| *Substance* | $R^2$ |
|---|---|
| 4-aminobiphenyl | 0.52 |
| 3-aminobiphenyl | 0.33 |
| 2-naphthylamine | 0.19 |
| *O*-toluidine | 0.20 |
| *M*-toluidine | 0.09 |
| *P*-toluidine | 0.15 |
| 2,4-dimethylaniline | 0.38 |
| 2,6-dimethylaniline | 0.52 |
| 2,3-dimethylaniline | 0.29 |
| 3,5-dimethylaniline | 0.13 |
| 3,4-dimethylaniline | 0.24 |
| 2,5-dimethylaniline | 0.31 |
| 3-ethylaniline | 0.26 |
| 2-ethylaniline | 0.31 |

### Discussion

We previously observed that the type and amount of tobacco smoked were associated with haemoglobin adduct levels formed by a few aromatic amines, particularly 4-aminobiphenyl (Bryant *et al.*, 1988). This observation was relevant, because 4-ABP is a powerful bladder carcinogen, and smokers, particularly of black, air-cured tobacco, are at high risk of bladder cancer (Vineis *et al.*, 1984). However, we noticed that the inter-individual variability in adduct levels unexplained by type and amount of tobacco smoked was considerable (between 84% and 46% according to the substance). It must be stressed that the proportion of variability explained by smoking habits, as reported in Table II, is likely to be an overestimation since the model is saturated.

We performed, therefore, an analysis of residual variability. Our hypothesis was that individual metabolic differences, as expressed by the correlation among adducts formed by different aromatic amines, could further explain part of the inter-individual variability. The results are clearly in agreement with such a hypothesis, since the correlation among a group of residuals explains 49% of the total variance of residuals, and the correlation among residuals of a second group explains a further 16% (Table IV). Moreover, the variables contributing to the first factor are all mononuclear aromatic amines, while those related to the second factor are binuclear amines (3-ABP, 4-ABP, 2-NA). Residuals of compounds from one group (e.g. mononuclear) are highly correlated to residuals of the other amines within the same group but are independent from residuals of compounds of the other group. In other words, subjects who have levels of one arylamine higher than expected have higher levels of the other compounds belonging to the same structural group too.

One possible interpretation of this finding is that two different metabolic pathways are involved, one for mononuclear and the other for binuclear aromatic amines, and that inter-individual variability is present for each of this pathways. The possibility that the levels of haemoglobin or DNA adducts in humans are influenced by metabolic patterns (including activation and deactivation of chemicals and DNA repair) has already been put forward. For example, Nowak *et al.* (1988) recently reported that, among 86 first-degree relatives of 15 families, inter-familial variations for benzo(a)pyrene DNA adducts were higher than intrafamilial variations, thus suggesting a genetic component. The activities of enzymes, such as hydrocarbon hydroxylase (Guengerich, 1988) or *N*-acetyltransferase (Schulte, 1988), involved in the activation or deactivation of carcinogenic compounds are known to be genetically determined, in the sense that genotypic polymorphisms exist in human populations. Acquired inter-individual differences are also possible, due to enzyme induction.

In the case of aromatic amines, it is plausible that different

**Table III** Pearson correlation coefficients between the residuals of the 14 aromatic amines after allowing for tobacco smoking habits[a]

| | 4-ABP | 3-ABP | 2-NA | O-TOL | M-TOL | P-TOL | 2,4-DIMET | 2,6-DIMET | 2,3-DIMET | 3,5-DIMET | 3,4-DIMET | 2,5-DIMET | 3-ETHYL | 2-ETHYL |
|---|---|---|---|---|---|---|---|---|---|---|---|---|---|---|
| 4-ABP[b] | 1 | 0.45† | 0.29* | 0.18 | 0.24 | 0.05 | −0.06 | 0.26 | 0.04 | 0.07 | 0.09 | 0.03 | 0.03 | 0.14 |
| 3-ABP | | 1 | 0.42† | 0.14 | 0.18 | 0.43† | 0.10 | 0.09 | −0.08 | 0.05 | 0.11 | −0.12 | 0.20 | 0.05 |
| 2-NA | | | 1 | 0.20 | 0.12 | 0.40† | −0.05 | −0.17 | −0.13 | −0.11 | 0.06 | −0.21 | 0.05 | −0.16 |
| O-TOL | | | | 1 | 0.57† | 0.34* | 0.49† | 0.11 | 0.39† | 0.35* | 0.39† | 0.47† | 0.43† | 0.56† |
| M-TOL | | | | | 1 | 0.61† | 0.52† | 0.48† | 0.71† | 0.72† | 0.75† | 0.58† | 0.79† | 0.54† |
| P-TOL | | | | | | 1 | 0.46† | 0.16 | 0.34* | 0.53† | 0.65† | 0.18 | 0.64† | 0.15 |
| 2,4-DIMET | | | | | | | 1 | 0.10 | 0.42† | 0.71† | 0.73† | 0.42† | 0.72† | 0.47† |
| 2,6-DIMET | | | | | | | | 1 | 0.68† | 0.43† | 0.37† | 0.60† | 0.44† | 0.52† |
| 2,3-DIMET | | | | | | | | | 1 | 0.71† | 0.71† | 0.93† | 0.78† | 0.80† |
| 3,5-DIMET | | | | | | | | | | 1 | 0.94† | 0.63† | 0.92† | 0.60† |
| 3,4-DIMET | | | | | | | | | | | 1 | 0.59† | 0.92† | 0.56† |
| 2,5-DIMET | | | | | | | | | | | | 1 | 0.69† | 0.93† |
| 3-ETHYL | | | | | | | | | | | | | 1 | 0.64† |
| 2-ETHYL | | | | | | | | | | | | | | 1 |

[a]For the method of computation of residuals see text. [b]For the meaning of abbreviations see Table II. $*P<0.05$; $\dagger P<0.01$. Correlations are: columns 2 and 3 with rows 1 and 2, between two different binuclear amines; columns 4–14 with rows 1–3, between one mononuclear and one binuclear amine; all others, between two different mononuclear amines.

**Table IV** Principal components analysis of the residuals of the 14 aromatic amines

| Substance | Factor 1 | Factor 2 | Factor 3 | Factor 4 |
|---|---|---|---|---|
| 4-ABP[a] | 0.05 | 0.29 | 0.58 | 0.02 |
| 3-ABP | 0.05 | 0.50 | 0.23 | −0.16 |
| 2-NA | 0.00 | 0.51 | 0.08 | 0.18 |
| O-TOL | 0.22 | 0.12 | 0.04 | 0.72 |
| M-TOL | 0.33 | 0.12 | 0.05 | −0.00 |
| P-TOL | 0.22 | 0.39 | −0.22 | −0.19 |
| 2,4-DIMET | 0.27 | 0.06 | −0.37 | 0.16 |
| 2,6-DIMET | 0.22 | −0.16 | 0.45 | −0.38 |
| 2,3-DIMET | 0.33 | −0.20 | 0.14 | −0.07 |
| 3,5-DIMET | 0.34 | −0.02 | −0.18 | −0.20 |
| 3,4-DIMET | 0.34 | 0.08 | −0.21 | −0.17 |
| 2,5-DIMET | 0.31 | −0.28 | 0.18 | 0.14 |
| 3-ETHYL | 0.36 | 0.06 | −0.15 | −0.14 |
| 2-ETHYL | 0.30 | −0.22 | 0.21 | 0.30 |
| Proportion of total variance explained by each factor | 49% | 16% | 10% | 7% |

Weights of residuals of each substance in the first four factors, see text for definition of factors and for the meaning of weights. [a]See Table II for the meaning of abbreviations.

metabolic pathways exist for mono- and binuclear compounds respectively. In particular, a single cytochrome P-450 is considered now to be responsible for *N*-hydroxylation of binuclear aromatic amines (Butler *et al*., 1990). Experiments have been conducted to determine the inducibility of covalent binding by continuous adminstration of either 2,6-dimethylaniline or 2-acetylaminofluorene to rats (Short & Hardy, 1989). An increase in covalent binding in the target organ was seen for 2,6-dimethylaniline while a decrease in the target organ was observed for 2-acetylaminofluorene. This can be interpreted as a difference in the type of metabolism induced by the monocyclic versus the policyclic aromatic amines and supports the hypothesis that there are different enzyme systems for these two classes of compounds.

The following alternative hypotheses can be considered.

1. Misclassification of the number of cigarettes smoked. In fact the reported number of cigarettes tends to concentrate on the values of 15 and 20. However, this phenomenon should have given, as a result, an artifactual correlation of residuals of all amines; it cannot explain the existence of two groups of compounds with different behaviours.

2. Exposure to sources of aromatic amines other than tobacco smoking. However, none of the subjects was clearly exposed to aromatic amines for occupational or other reasons other than smoking. In addition, in order to justify this interpretation some subjects should have been exposed to mononuclear amines only and others to binuclear amines only, which is totally implausible.

3. Measurement errors might be reciprocally correlated within, but not between, the two groups of substances. This, however, seems to be a very implausible explanation of the findings, considering that many correlation coefficients are higher than 0.5 and a few are higher than 0.9. In order to justify a correlation coefficient of 0.5, variance due to measurement error should be as large as the true variance of adduct concentrations, and to justify a correlation coefficient of 0.9 it should be nine times as large. Clearly this is very unlikely.

In conclusion, none of the considered alternative hypotheses seems to be able to explain completely the findings, even if they could account for part of them. On the basis of the present results it is plausible that inter-individual metabolic differences affect internal levels of exposure to carcinogens. Such metabolic patterns might be relevant to the individual risk of cancer. However, this suggestion needs to be verified in further epidemiological and experimental studies.

We thank Drs Fred Kadlubar, Benedetto Terracini and Neil Caporaso for thoughtful advice. This research was supported by the Associazione Intaliana per la Ricerca sul Cancro, by the Italian National Research Council (Progetto Finalizzato Oncologia, grant 86.005.95.44) and by PHS grant no. ES00597, awarded by the USA National Institute of Environmental Health Services. Technical support was provided by CSI Piemonte (computing devices). We are indebted to AVIS (Associazione Italiana Volontari del Sangue) and Dr Fisso for fruitful co-operation.

### References

BUTLER, M.A., IWASAKI, M., GUENGERICH, F.P. & KADLUBAR, F.F. (1990). Human cytochrome $P450_{PA}$ ($P450_{IA2}$), the phenacetin O-deethylase is primarily responsible for the hepatic 3-demethylation of caffeine and the N-oxidation of carcinogenic arylamines. *Proc. Natl Acad. Sci. USA* (in the press).

BRYANT, M.S., VINEIS, P., SKIPPER, P.L. & TANNENBAUM, S.R. (1988). Haemoglobin adducts of aromatic amines: association with smoking status and type of tobacco. *Proc. Natl Acad. Sci. USA*, **85**, 9788.

GUENGERICH, F.P. (1988). Roles of cytochrome P-450 enzymes in chemical carcinogenesis and cancer chemotherapy. *Cancer Res.*, **48**, 2946.

IARC (1987). Monographs on the evaluation of carcinogenic risk to humans. Overall evaluation of carcinogenicity: an updating of IARC Monographs Volumes 1 to 42. Supplement 7. IARC Scientific Publications: Lyon.

KLEINBAUM, D.G. & KUPPER, L.L. (1978). *Applied Regression Analysis and Other Multivariable Methods*. Duxbury Press: Boston.

NOWAK, D., SCHMIDT-PREUSS, U., JORRES, R., LIEBKE, F. & RUDIGER, H.W. (1988). Formation of DNA adducts and water soluble metabolites of benzo(a)pyrene in human monocytes is genetically controlled. *Int. J. Cancer*, **41**, 169.

SAS INSTITUTE (1987). *SAS User's Guide: Statistics*, 1987 Edition. SAS: Cary, NC.

SHORT, C.R. & HARDY, M.L. (1989). Covalent binding of [$^{14}$C]-2, 6-dimethylaniline to DNA of rat liver and ethmoid turbinate. *J. Toxicol. Environ. Health*, **27**, 85.

SCHULTE, P.A. (1988). The role of genetic factors in bladder cancer. *Cancer Detect. Prev.*, **11**, 379.

VINEIS, P., ESTÈVE, J. & TERRACINI, B. (1984). Bladder cancer and smoking in males: types of cigarettes, age at start, effect of stopping and interaction with occupations. *Int. J. Cancer*, **34**, 165.